# Optimizing for taxonomic coverage: a comparison of methods to recover mesofauna from soil

**DOI:** 10.21307/jofnem-2020-104

**Published:** 2020-10-21

**Authors:** Alexandros Dritsoulas, Larry W. Duncan

**Affiliations:** Citrus Research and Education Center (CREC), Institute of Food and Agriculture Sciences (IFAS), University of Florida (UF), 700 Experiment Station Road, FL, 33850

**Keywords:** Entomopathogenic nematodes, Heptane floatation, Microarthropods, Soil food web, Sucrose centrifugation, Tullgren funnels

## Abstract

Manipulating soil properties to modify the dynamics between nematodes and their natural enemies has been proposed to conserve services such as the biological control of insect pests by entomopathogenic nematodes. Many soil microarthropods including acari mites and collembola are natural enemies of nematodes; however, little is known about the naturally occurring assemblages of these two soil dwelling groups and how they might be influenced by soil conditions. A method to efficiently recover both nematodes and microarthropods from environmental samples would be helpful to characterize communities of these two groups in different habitats. Because samples of nematodes extracted from soil by sucrose centrifugation (SC) also contain soil mites, collembola, protozoans, and fungal and bacterial propagules, the efficiency of SC to recover microarthropods was compared to more conventional methods of microarthropod recovery such as heptane flotation (HF), Berlese funnels (BF), and a modified flotation Berlese method (FBF). Microarthropods were identified using an inverted microscope to class in one experiment and to order in a second. Significantly more microarthropods of all taxa were recovered by SC than with either Berlese method (BF or FBF). In total, 40% more microarthropods comprising seven orders were recovered by HF compared to SC, but the difference was not significant. Ecological indices (diversity, richness, and evenness) derived from HF and SC were congruent and significantly higher than those derived from BF. Excessive organic matter in the HF extractions, compared to those of SC, BF, and FBF, made mite detection and identification difficult and time consuming. Moreover, unlike SC, neither HF nor any Berlese method recovered nematodes. Accordingly, we found SC to be the most efficient method for microarthropod extraction, making it an ideal method for studies of communities of nematodes and many of their natural enemies in the soil.

Nematodes occupy trophic levels in a food web that includes natural enemies such as nematophagous fungi (Kaya and Koppenhöfer, 1996), ectoparasitic bacteria (Enright and Griffin, 2005; El-Borai et al., 2005), and soil microarthropods (Walter and Ikonen, 1989). Mites, springtails, and other microarthropods are major components of soil biodiversity and food web function. Numerous reports indicate that these generalist predators dominate the higher trophic levels that regulate virtually all nematode populations. For example, the mesostigmatid mite *Protogamasellus mica* was shown to consume bactivorous, fungivorous, and phytophagous nematode species at approximately the same rate regardless of the size or motility of its prey (Stirling et al., 2017). As such, microarthropods are among a diverse guild of soil organisms that attenuates processes such as crop loss to plant parasitic nematodes (Joharchi et al., 2015; Yang et al., 2020) and biological control of crop pests by entomopathogenic nematodes (Poinar, 1979; Epsky et al., 1988; Walter and Ikonen, 1989; Karagoz et al., 2007).

Despite the agricultural and ecological implications of understanding how nematode and microarthropod populations affect one another, information from laboratory and greenhouse research is supplemented by relatively few field studies (Santos and Whitford, 1981; Wilson and Gaugler, 2004; Jabbour and Barbercheck, 2011; Bal et al., 2017). Geospatial and temporal surveys of the combined, natural occurrence of nematodes and microarthropods are rare (Duncan et al., 2007), but will likely increase as affordable metagenomic tools provide wider opportunity to study cryptic soil communities. Relationships measured between biotic and abiotic variables in soil food web surveys can reveal potential key natural enemies of nematodes as well as cultural practices that manipulate the soil in ways to exploit the services of biological control agents of harmful nematodes and arthropod pests (Duncan et al., 2013; Campos-Herrera et al., 2015, 2019). For example, surveys of naturally occurring communities of entomopathogenic nematodes and some of their natural enemies have identified soil properties such as pH (Hara et al., 1991; Campos-Herrera et al., 2013a), salinity (Hara et al., 1991; Nielsen et al., 2011), texture and moisture (Campos-Herrera et al., 2013b) that potentially modulate EPN populations directly, or indirectly by affecting their hosts (Gazit et al., 2000) or natural enemies (Duncan et al., 2013; Campos-Herrera et al., 2019).

The study of nematodes and their natural enemies would be facilitated by a common method to extract both groups from the soil. Traditional methods of recovering organisms from soil vary by discipline. Estimates of optimum methods based on extraction efficiency and cost usually focused on one group of organisms among many that might be recovered. For the purpose of studying nematode and microarthropod communities, we are unaware of any comparisons of extraction efficiencies of both groups by a given extraction method. Microarthopods and nematodes can both be separated from soil by passive methods (flotation, rinsing, adhesion), or by allowing organisms to migrate from soil into a trapping device (Berlese funnels or Baermann funnels) (Southey, 1970; Krantz et al., 2009). Nematodes are most commonly recovered using various modifications of the Baermann funnel (Townshend, 1963) or centrifugal flotation (Jenkins, 1964). The most commonly used procedure to recover microarthropods is Tullgren extraction using Berlese funnels. In Tullgren extraction, the litter or soil samples are placed on a mesh screen inside a collection funnel. Light and heat is applied to the upper side of the sample, creating a temperature gradient which causes a progressive desiccation which drives microarthropods from the sample and into a collection vessel. Heptane flotation (HF) exploits the lipophilic nature of the microarthropods’ cuticle where the apolar waxy cuticle has affinity for the polar heptane and not to the apolar water (Aucamp and Ryke, 1964). The amount soil processed by these procedures is generally in the range of 100 to 250 g, but this volume is insufficient to capture arthropod diversity in the deeper, mineral soil fraction using Tullgren extraction. For this reason, Arribas et al. (2016) added an extra flotation step (described below) to the Tullgren extraction protocol in order to improve the efficiency of the Berlese in recovering microarthropods from the extraction substrate.

Mites and collembola are commonly encountered in nematode samples extracted by sucrose centrifugation. Duncan et al. (2007) measured food web responses to bare and manure-mulched soil augmented with entomopathogenic nematodes, where nematodes, mites, collembola, enchytraeid worms, nematophagous fungi, and bacterial ectoparsites of EPNs were all extracted with SC. Sucrose centrifugation is also used for extracting mycorrhizae, by virtue of the spores (Shamini and Amutha, 2014). Here, we compared the efficiency of SC to three methods developed for capturing microarthropods. Our hypothesis was that SC, unlike the other methods, is an efficient technique to recover both nematodes and microarthropods from mineral soil and is especially well-suited to ecological studies of nematodes and their natural enemies. Our objectives were to (i) evaluate microarthropod extraction efficiency of SC compared to that of a modified flotation–Berlese Funnel method, and (ii) compare the efficiency of SC, Berlese funnels and heptane flotation for characterizing microarthropod communities.

## Material and methods

### Sucrose centrifugation and flotation–Berlese–flotation comparison

We compared the microarthropod extraction efficiency of a flotation–Berlese–flotation (FBF) method (Arribas et al., 2016) to that of sucrose centrifugation (Jenkins, 1964; SF), using soil samples from an experimental citrus orchard adjacent to the University of Florida Department of Entomology and Nematology. An auger was used to extract 24 cores (dia. 10.5 cm  ×  23 cm depth; ~2000 ml volume). Two cores collected from each of 12 trees were processed by either FBF or SC.

**Figure 1:**
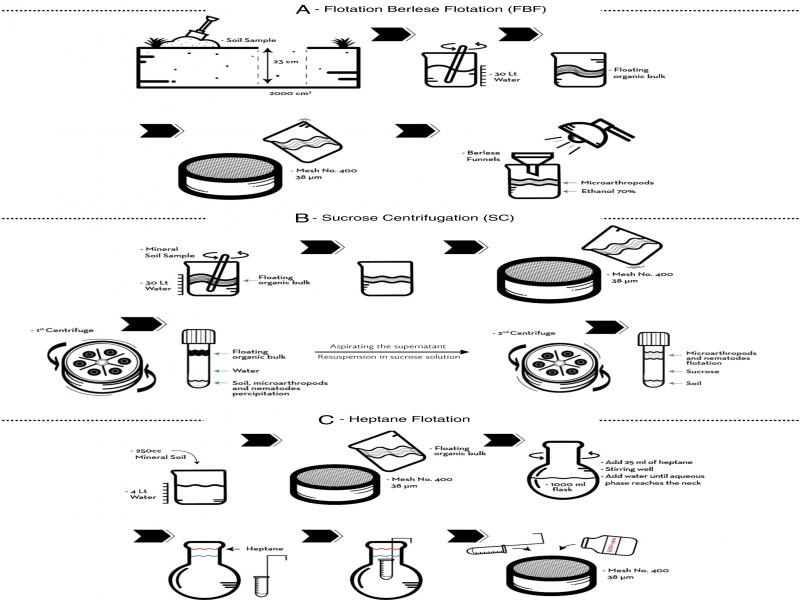
Process flow diagrams for four extraction methods. (A) Flotation–Berlese–flotation and simple Berlese device, (B) Sucrose centrifugation, and (C) Heptane flotation.

For FBF (Fig. 1A), the large 2-liter soil sample was mixed vigorously in a bucket with 30 liters of water to dissolve soil aggregates and allow mineral material to sediment. Immediately after mixing, the floating material was filtered through a 400-mesh sieve (38 microns) to obtain a bulk subsample of < 250cc containing organic matter and soil mesofauna. Subsamples were processed in a Berlese apparatus (Berlese, 1905; Tullgren, 1918; Southwood and Henderson, 2000), where the sample rested on a layer of cheese-cloth placed over a plastic mesh in the 30.5 cm dia. funnel and exposed to a moderate vertical gradient of heat and light (25 watt lamp) until the organic material was completely dry (approx. 5 days). All mesofauna collected in the flask of 95% alcohol beneath the funnel were captured on a 400-mesh sieve and preserved in absolute ethanol in a 15 ml tube.

Samples extracted by SC (Fig. 1B) were processed initially in the same manner as for FBF, except that the organic subsample was collected in 2 to 4, 100-ml centrifuge tubes and centrifuged 1,700 rpm (810 g) to precipitate nematodes and soil particles and remove organic debris in the decanted supernatant. The samples were then resuspended in a high-density sugar solution (specific gravity = 1.10-1.18) to precipitate soil particles and suspend mesofauna in the supernatant for collection with a sieve.

Microarthropod specimens were identified at the level of subclass for Collembola and Acari mites using a dissecting microscope. Significant differences between sampling methods in the frequency of specimens were determined by *t*-test.

### Sucrose centrifugation, Berlese funnels, and heptane flotation comparison

Samples were taken from 15 sites (auger dimensions, dia. 2.5 cm × 28 cm depth) in the Natural Area adjacent to the Department of Entomology and Nematology. In total, 12 cores were taken at each site and four randomly chosen cores were combined into three subsamples of 250cc. One subsample from each of the 15 sites was processed either by BF, SC, or HF. However, samples processed with HF produced an excess of organic matter making detection of microarthropods difficult. Consequently, two data sets were created as two independent experiments. The efficiency of BF compared to that of SC was determined using data from 15 sites. A comparison of all three methods was made using data from just six of the sites from which counts were also made for the HF method. Nematodes were also counted in six samples from each extraction method.

Fauna were extracted in Berlese funnels described previously. The illumination of the apparatus was controlled by a potentiometer adjusted the first day to produce a weak vertical gradient of heat and dimmed light. The light brightness was increased chromatically each passing day and the samples remained in the funnels for 8 days.

Samples were processed by SC as in the previous experiment, except that the volume of organic matter was small enough to process each sample in a single tube.

To extract the microarthropods by HF (Fig. 1C), the 250cc sample of mineral soil was suspended in 4 liters water and then decanted through a 400-mesh sieve. The collected organic matter was washed into a round 1000 ml flask and resuspended in 500 ml. About 25 ml of heptane was added to the soil–water mixture, stirred for 2 min to allow the microarthropods to come into suspension in the heptane layer. Distilled water was slowly added until the heptane was in the neck of the flask. With a dipper-ladle, the organic phase and part of aqueous phase was collected and poured through a 400-mesh sieve. The collected fauna and organic matter were rinsed several times with 95% ethanol to remove the excess heptane and transferred to a vial in ethanol suspension. Microarthropod specimens were identified with light microscopy at the level of subclass for Collembola, order for Protura and Diplura, and suborder for Acari mites. Three ecological indices were estimated from the six samples common to each method: species richness (number of species, *S*); Shannon–Weaver diversity index, $H^{\prime }=-\sum_{n=1}^{s}p_{I}\log_{e}p_{I},$ where *pi* is the proportion of species *i* (Pielou, 1975); and Simpson’s (1949) index of dominance, D′$D^{\prime }=1-\sum_{n=1}^{s}p_{I}$ .

### Statistical analyses

Comparison of mean differences for BF and SC (*n* = 15) were by *t*-test. A Kruskal–Wallis test was used to assess significance of differences in means of the three extraction methods (*n* = 6) and Tukey’s honestly significant difference test (*P* = 0.05) was used to compare means of log_*n*_ *X* + 1 transformed data. Proportional representations of the means of each taxon were created using raw data and JMP^®^ (SAS institute) software. Differences in the ecological indices were also assessed by Kruskal–Wallis and Tukey HSD tests. The pairwise relationships between numbers of mites and Collembola extracted by BF, HF, and SC were measured using linear regression of log (*X* + 1) transformed numbers.

## Results

### Sucrose centrifugation and flotation–Berlese–flotation comparison

**Figure 2:**
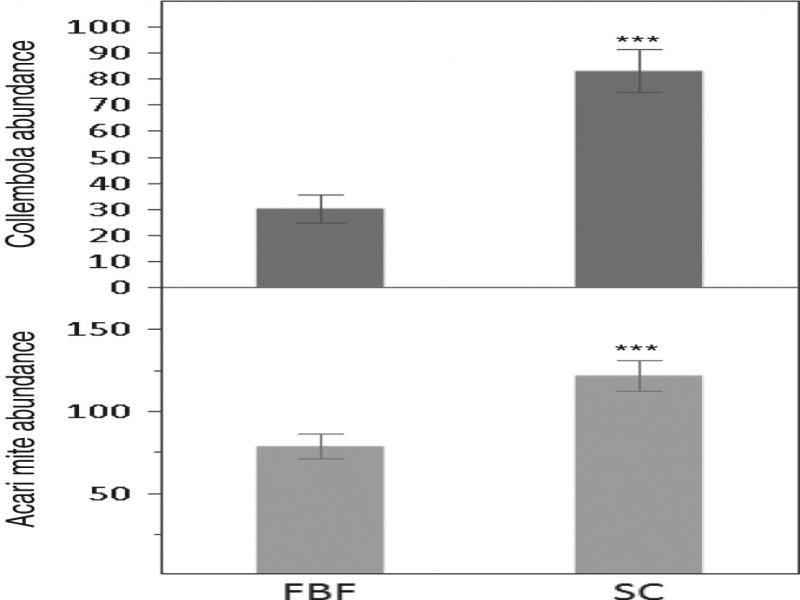
Efficiency of sucrose centrifugation (SC) compared to that of flotation–Berlese–flotation (FBF) for extracting Acari mites and Collembola from 2 L mineral soil samples. Differences between taxa abundance evaluated by *t*-test (*N* = 12, ***P < 0.001). Data presented as mean ± standard error.

The microarthropod extraction efficiency of SC was 88% higher than that of FBF in the first experiment. In total, 55% more mites (*t*_22_ = 3.64, *P* = 0.0014) and 177% more collembola (*t*_22_ = 5.33, *P* < 0.001) were recovered using SC than FBF (Fig. 2). No nematodes were detected in samples extracted by FBF. By contrast, extracting the relatively large soil sample by SC yielded abundant nematodes, which in some cases interfered with counting microarthropods.

### Sucrose centrifugation and Berlese funnel comparison

**Figure 3:**
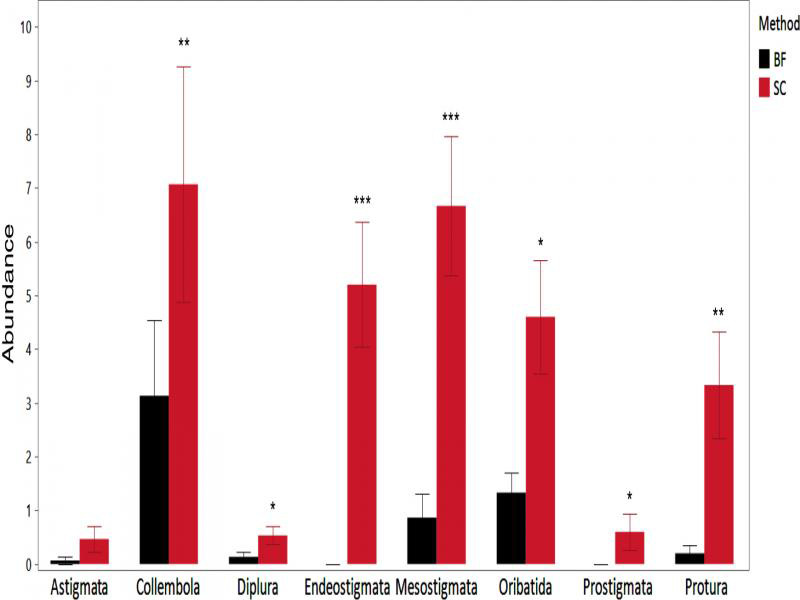
Efficiency of sucrose centrifugation (SC) compared to that of Berlese funnels (BF) in extracting eight microarthropod taxa from 250cc mineral soil sample. Wilcoxon nonparametric multiple comparisons used to test the differences between the methods. Data are presented as mean ± standard error test (*N* = 12; *P < 0.05; **P < 0.01; ***P < 0.001).

Approximately 20% as many microarthropods were recovered by BF compared SC in the second experiment (Fig. 3). Sucrose centrifugation was more efficient than BF in recovering Mesostigmata (*W* = 13.24, df = 1, *P* < 0.001), Prostigmata (*W* = 5.77, df = 1, *P* = 0.016), Oribatida (*W* = 5.89, df = 1, *P* = 0.015), Endeostigmata (*W* = 19.99, df = 1, *P* < 0.001), Protura (*W* = 10.32, df = 1, *P* = 0.001), Diplura (*W* = 3.99, df = 1, *P* = 0.046), and Collembola (*W* = 7.04, df = 1, *P* = 0.008). Only Astigmata (*W* = 2.26, df = 1, *P* = 0.13) were not significantly more abundant in the samples extracted by SC. The SC samples contained abundant nematodes, comparable to our repeated experience in sampling this locality for classroom teaching, whereas neither FBF nor BF recovered any nematodes.

### Sucrose centrifugation, Berlese funnel, and heptane flotation comparison

**Figure 4:**
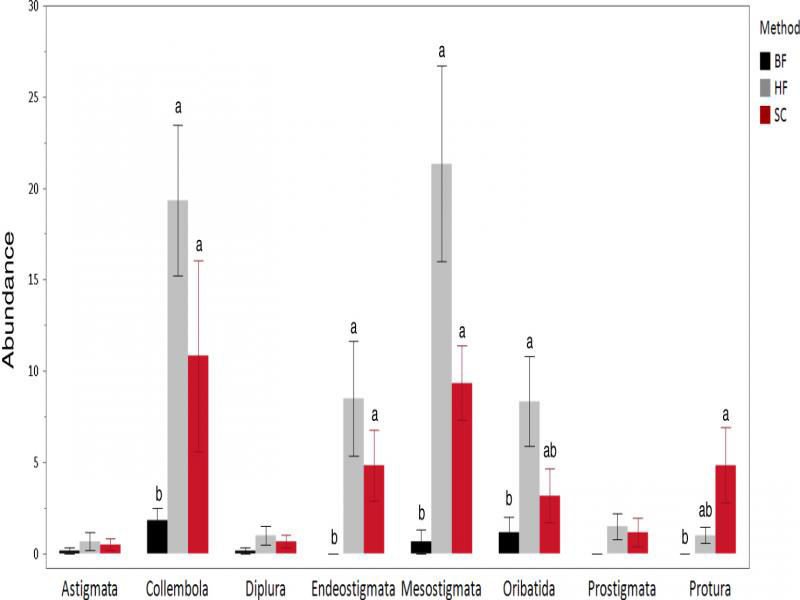
Extraction efficiency of Berlese funnels (BF), sucrose centrifugation (SC), and heptane flotation (HF). Bars and error bars denote means and 95% confidence intervals respectively. Kruskal–Wallis test was used to evaluate differences between the three different methods for each taxa Tukey–Kramer method was employed for the mean comparisons which are represented by different small letters above bars.

In the third experiment in which subsamples from just 6 of the 15 sites sampled in experiment 2 were extracted, consistently more microarthropods were recovered by HF than by SC or BF (Fig. 4). Berlese funnels recovered fewer animals than HF in five of the eight taxa and fewer than both other methods in four taxa. This was reflected by comparing the three different methods with Kruskal–Wallis test for each taxon; specifically, Mesostigmata (*χ*^2^ = 12.31, df = 2, *P* = 0.002), Prostigmata (*χ*^2^ = 4.075, df = 2, *P* = 0.130), Oribatida (*χ*^2^ = 7.288, df = 2, *P* = 0.026), Endeostigmata (*χ*^2^ = 10.71, df = 2, *P* = 0.005), Protura (*χ*^2^ = 9.44, df = 2, *P* = 0.009), Diplura (*χ*^2^ = 2.177, df = 2, *P* = 0.337), Astigmata (*χ*^2^ = 0.6977, df = 2, *P* = 0.706), and Collembola (*χ*^2^ = 9.956, df = 2, *P* = 0.007). Although HF recovered larger numbers of microarthropods than did SC for seven of the taxa, and twice as many overall, the differences were not significant. Linear regressions (*N* = 48) of the numbers of mites of each taxon recovered by each method resulted in no relationship between BF and HF (*R*^2^ = 0.069, *F*_1,46_ = 3.39, *P* = 0.072), a weak positive trend for BF and SC (*R*^2^ = 0.096, *F*_1,46_ = 4.86, *P* = 0.0325), and a strong relationship between HF and SC (*R*^2^ = 0.29, *F*_1,46_ = 19.15, *P* < 0.0001). In particular, BF failed to recover prostigmatids and especially endeostigmatids that comprised 14% of communities recovered by both HF and SC.

**Figure 5:**
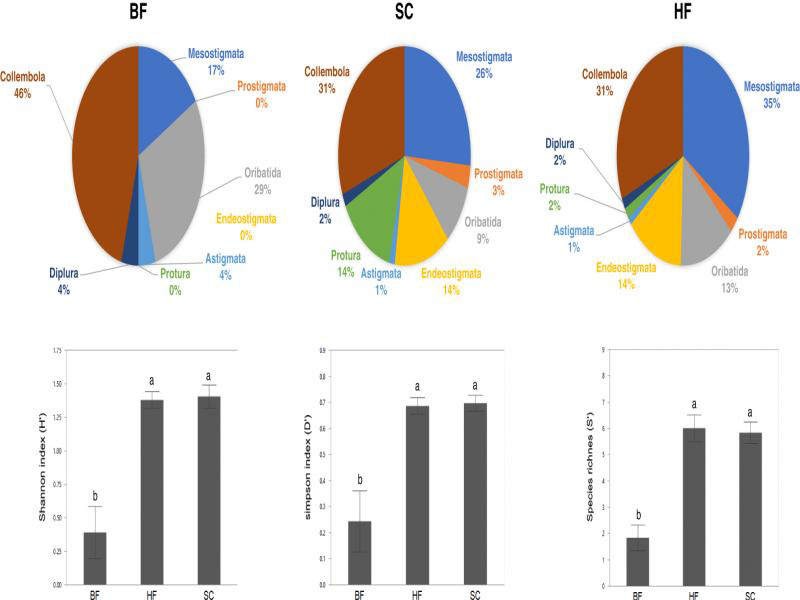
Pie charts: proportional composition of microarthropod taxa. Pie charts represent recovery from Berlese funnels (BF), sucrose centrifugation (SC), and heptane flotation (HF). Barplots: ecological indices (species richness, S′; Shannon diversity index, H′; dominance, D′) from samples extracted by three methods, Berlese funnels (BF), sucrose centrifugation (SC), and heptane flotation (HF). Bars and error bars denote means and 95% confidence intervals. Means that are significantly different in multiple comparison using Tukey’s HSD test are represented by different small letters above bars (*P* < 0.05).

The ecological indices S′, H′, and D′ estimated with data from HF and SC were congruent, whereas those from BF were lower in all cases. Kruskal–Wallis was performed using the software R (R Development Core Team, ‘dplyr’ package; S′, *χ*^2^ = 11.421, df = 2, *P* = 0.003; H′, *χ*^2^ = 10.864, *P* = 0.004; D′, *χ*^2^ = 10.194, *P* = 0.006) (Fig. 5). Just two individual nematodes were observed in all six samples processed by HF and no nematodes were observed using BF. By contrast 1623 ± 223 nematodes per 250 cc soil (mean and standard error) were recovered by SC.

## Discussion

A variety of methods exist to extract nematodes from soil, all of which differ in the efficiency with which they recover different species (Southey, 1970). To our knowledge, no extraction method has been recommended for recovery of both microarthropods and nematodes (McSorley and Walter, 1991). Of the four methods studied here, those used primarily by acarologists recovered virtually no nematodes. The two nematode individuals recovered by HF were likely trapped in the copious organic aggregates recovered by the method. By contrast, the widely used nematode extraction method was surprisingly effective in recovering microarthropods.

According to Petersen and Luxton (1982), passive extraction methods are more efficient than active methods such as Berlese funnels. Nevertheless, Berlese funnels are the most frequently used extraction method for soil microarthropods. Over 90% of selected acarology studies utilized Berlese funnels, despite reportedly poor efficiency in recovering certain taxa and immature stages (Andre et al., 2017). Most of those studies restricted sample depth to 10 cm, because of low recovery efficiency in mineral soil compared to flotation methods (Ducarme et al., 1998). This is consistent with our finding that BF recovered about 20% as many microarthropods as did SC and even fewer than HF.

The inefficiency of Berlese funnels for recovery of microarthropods in deeper soil layers resulted in numerous modifications such as the FBF technique. Flotation–Berlese–flotation separates animals and organic matter from a large volume of mineral soil, and then relies on motility to recover just the microarthropods (Arribas et al., 2016). Consequently, in comparison to SC, FBF still suffers from the poor extraction efficiency of the simple Berlese funnel method. Moreover, FBF requires large (2000 ml) amounts of soil, which creates practical problems in handling the samples compared to other methods.

Despite shortcomings, Berlese funnels produce clean samples for microscopy and molecular processing. The large quantity of organic debris in the HF product is a serious impediment, despite the exceptionally high recovery of animals with this technique. The excessive impurities not only make identification and counting laborious and time consuming, they are likely to interfere with using toolkits for DNA extraction.

The consistently higher numbers of microarthropods captured by HF compared to SC suggest that the lack of significant differences was due to inadequate replication. Nevertheless, the relative recovery of the various taxa by HF and SC was highly correlated, and the community structure and diversity reflected by the two methods were nearly identical. This analytical congruence, combined with cleaner samples that facilitate counting or molecular analysis and the fact that only SC will recover nematodes, make it an ideal extraction method for studying both groups of animals. Moreover, the loss of saprophytic soil fungal and bacterial propagules during the sieving and rinsing process of SC is advantageous because primarily fungi and bacteria intimately associated with nematodes, mites, and collembola are retained. This property has been exploited in studies that utilized qPCR tools to estimate the occurrence and dynamics of fungal and bacterial natural enemies of entomopathogenic nematodes (Campos-Herrera et al., 2014; Pathak et al., 2017). Sucrose centrifugation appears to be uniquely suited to study species assemblages affecting soil nematodes, given the breadth of natural enemy guilds it can capture.
